# External validation of an artificial intelligence tool for fracture detection in children with osteogenesis imperfecta: a multireader study

**DOI:** 10.1007/s00330-025-11790-z

**Published:** 2025-07-07

**Authors:** Cato Pauling, Harsimran Laidlow-Singh, Emily Evans, David Garbera, Rosalind Williamson, Ranil Fernando, Kate Thomas, Helena Martin, Owen J. Arthurs, Susan C. Shelmerdine

**Affiliations:** 1 University College London, Gower Street, London, UK; 2 Department of Paediatric Radiology, Evelina London Children’s Hospital, Guy’s and St Thomas NHS Foundation Trust, London, United Kingdom; 3 Department of Clinical Radiology, University Hospital Coventry and Warwickshire, Coventry, UK; 4 Department of Paediatric Radiology, Alder Hey Children’s NHS Foundation Trust Hospital, Liverpool, UK; 5 Victoria Hospital, NHS Fife, Kirkcaldy, UK; 6 Guy’s and St Thomas’ NHS Foundation Trust, London, UK; 7 Great Ormond Street Hospital for Children, London, UK; 8 UCL Great Ormond Street Institute of Child Health, University College London, London, UK; 9 Great Ormond Street Hospital NIHR Biomedical Research Centre, London, UK

**Keywords:** Artificial intelligence, Children, Fracture, Osteogenesis imperfecta, Radiography

## Abstract

**Objective:**

To determine the performance of a commercially available AI tool for fracture detection when used in children with osteogenesis imperfecta (OI).

**Materials and methods:**

All appendicular and pelvic radiographs from an OI clinic at a single centre from 48 patients were included. Seven radiologists evaluated anonymised images in two rounds, first without, then with AI assistance. Differences in diagnostic accuracy between the rounds were analysed.

**Results:**

48 patients (mean 12 years) provided 336 images, containing 206 fractures established by consensus opinion of two radiologists. AI produced a per-examination accuracy of 74.8% [95% CI: 65.4%, 82.7%], compared to average radiologist performance at 83.4% [95% CI: 75.2%, 89.8%]. Radiologists using AI assistance improved average radiologist accuracy per examination to 90.7% [95% CI: 83.5%, 95.4%]. AI gave more false negatives than radiologists, with 80 missed fractures versus 41, respectively. Radiologists were more likely (74.6%) to alter their original decision to agree with AI at the per-image level, 82.8% of which led to a correct result, 64.0% of which were changing from a false positive to a true negative.

**Conclusion:**

Despite inferior standalone performance, AI assistance can still improve radiologist fracture detection in a rare disease paediatric population. Radiologists using AI typically led to more accurate diagnostic outcomes through reduced false positives. Future studies focusing on the real-world application of AI tools in a larger population of children with bone fragility disorders will help better evaluate whether these improvements in accuracy translate into improved patient outcomes.

**Key Points:**

***Question*** *How well does a commercially available artificial intelligence (AI) tool identify fractures, on appendicular radiographs of children with osteogenesis imperfecta (OI), and can it also improve radiologists’ identification of fractures in this population?*

***Findings*** *Specialist human radiologists outperformed the AI fracture detection tool when acting alone; however, their diagnostic performance overall improved with AI assistance.*

***Clinical relevance*** *AI assistance improves specialist radiologist fracture detection in children with osteogenesis imperfecta, even with AI performance alone inferior to the radiologists acting alone. The reason for this was due to the AI moderating the number of false positives generated by the radiologists.*

**Graphical Abstract:**

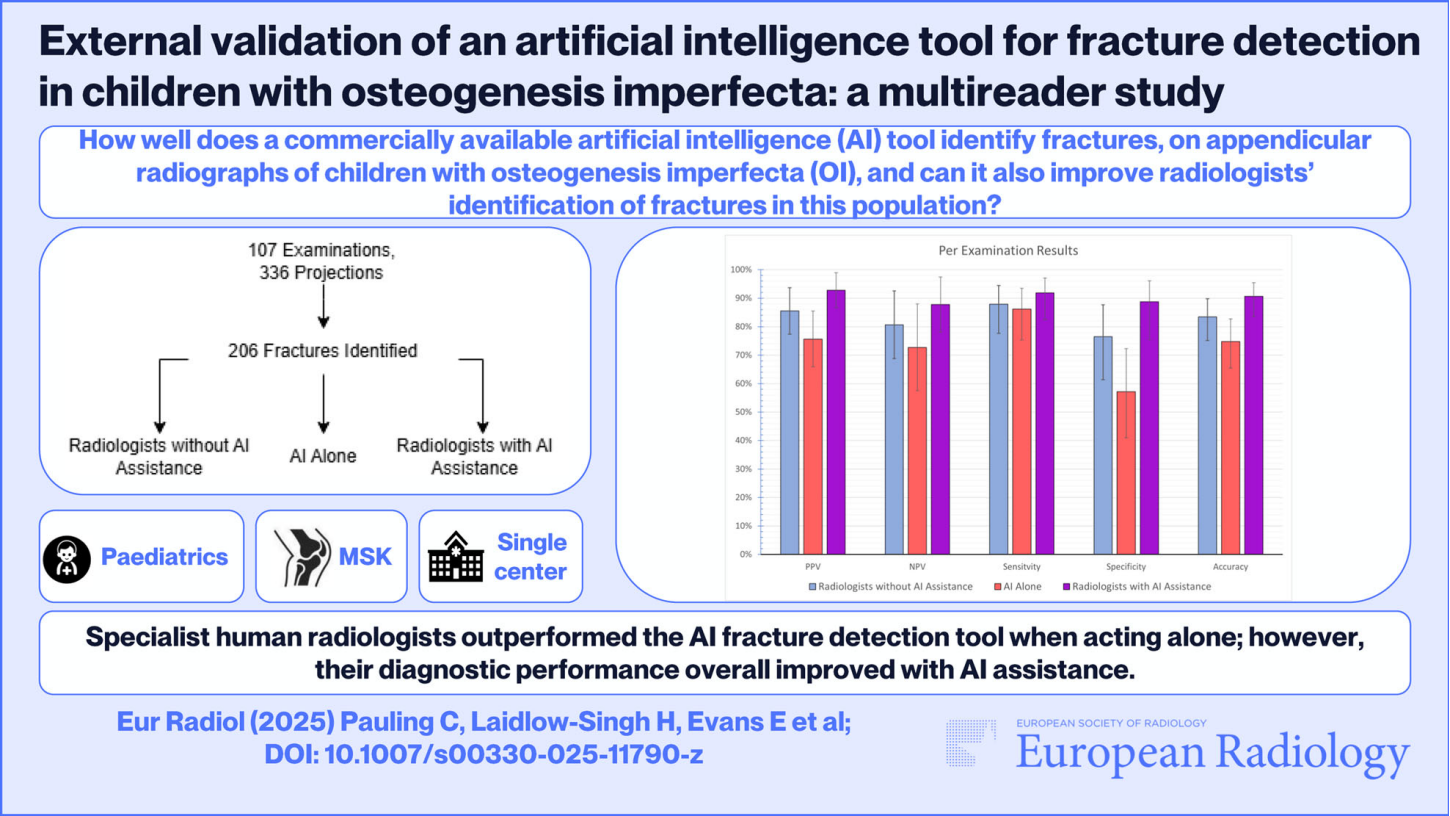

## Introduction

Osteogenesis imperfecta (OI) is a genetic condition characterised by skeletal fragility with an increased risk of fractures [1, 2]. It affects approximately 1 in 10,000 individuals [3], and many sufferers contend with chronic disability and pain from these repeated injuries. There are several different subtypes with variation in fracture predisposition, but the commonest types (I and IV) are often identified by multiple fractures during early childhood, together with genetic testing. As their bones can appear unusual after repeated fractures, healing and refracturing, new fractures can be easily missed by healthcare professionals, particularly those with insufficient experience with this rare condition.

Artificial intelligence (AI) is increasingly used to aid clinicians in the diagnosis of fractures on conventional radiographic imaging [4–9]. Meta-analyses have reported that AI assistance for fracture detection can increase diagnostic performance of healthcare professionals [6–9], although those evaluating children and rare disease populations remain distinctly lacking [10, 11]. Given that commercial fracture detection AI tools are now being integrated into clinical practice across different institutions worldwide, the unknown performance of such algorithms in paediatric and rare disease fracture detection is critical to better understand and safeguard against sources of patient discrimination and bias when implementing AI tools.

The purpose of this study was therefore to evaluate whether a commercially available AI fracture detection tool could improve radiologists’ detection of skeletal fractures in children with a rare disease, osteogenesis imperfecta (OI). This tool has not been specifically trained in populations with a bone fragility disorder; however, our intention was to measure potential performance or risks of implementation without adaptation.

## Methods

### Ethical approval

Ethical approval was provided by the National Health Service (NHS) Health Research Authority (HRA) (IRAS ID: 274278, REC reference 22/PR/0334). Our methodology follows the latest CLAIM [12] guidelines.

### Imaging case selection

A retrospective review of all consecutive appendicular and pelvic radiographs at Great Ormond Street Hospital for Children was performed over a 13-month study period (1/4/2021–30/4/2022) for children < 18 years of age with genetically confirmed diagnosis of osteogenesis imperfecta (OI).

Radiographs were extracted and anonymised from the local PACS system and uploaded to a cyber-secure, image viewing, multireader platform (Collective Minds Radiology®). Additional details are in the Electronic supplementary material.

### Data collection and labelling

The ground truth was determined by a consensus opinion of two consultant paediatric radiologists (with > 10 years of paediatric radiology experience each), with knowledge of the clinical indication and availability of prior examinations, where present. If a fracture was present, the abnormal region on the radiograph was highlighted with a bounding box and labelled as either acute or healing (i.e., healing was defined as the presence of a callus or periosteal reaction, with visible residual fracture line). Where there was no longer a visible fracture line, the bone was considered to be ‘remodelled’, and this was not labelled as a fracture.

### Multireader trial design

Seven radiologists (5 consultants (each with > 5 years paediatric radiology experience), 2 trainees (each with 6 months paediatric radiology experience)), with a special interest in paediatric radiology, were recruited from 5 different institutions across the UK. None had reported any of the OI cases in our multi-case dataset, and we did not provide the radiologists with any clinical information or prior images for comparison, apart from informing them of the study design. Radiographic examinations in this study were initially extracted as DICOM files from the hospital PACS System. DICOM tags that contained any patient identifiable information (e.g., name, hospital number, age, sex, weight, address, etc.) were removed, leaving only relevant technical information regarding the image and mode of acquisition (e.g., resolution, pixel size) in order to allow for AI analysis.

The multireader study was conducted in two separate rounds, with individual radiologists acting as their own controls across the rounds of image interpretation. Definitions for acute and healing fractures were discussed, and an online tutorial on how to use the imaging platform was provided to all radiologists prior to the study. The first reading round (30/11/2022 to 30/1/2023) involved the radiologists independently reviewing and annotating relevant radiographs with bounding boxes where a fracture was suspected, which included labelling of the fracture as acute or healing.

An interval washout period of 8 weeks was then ensured to avoid any recall. In the second round (16/4/2023 to 16/6/2023), radiologists were assisted by AI. The same randomly re-ordered set of images was re-annotated by the radiologists with the output of the AI tool (Milvue Suite, SmartUrgences®) available for guidance. The output generated by the AI included bounding boxes around areas of high probability of fracture. However, the AI did not have the capability to describe the fractures as acute or healing. Radiologists were asked to look at the AI output before re-annotating the same images again.

### Interventions

Milvue Suite-SmartUrgences® (v1.26) is a CE marked MDR Class 2a medical device, developed and commercialised by Milvue. The training and development of the AI tool has already been published elsewhere in detail [13–15]; but briefly, it was conducted on a multicentric dataset of more than 600,000 chest and musculoskeletal radiographs across seven key pathologies (fracture, pleural effusion, lung opacification, joint effusion, lung nodules, pneumothorax, and joint dislocation). While the exact population used for the training of this model is not public, personal correspondence with the vendor informs us that the proportion of the training dataset dedicated to children included 8.2% aged 0–12 years old and 13% aged 13–25 years old. The vendor confirms the product is intended for adult and paediatric use, with no defined lower age limit.

For each positive finding, the AI provides a binary certainty score (that is, certain/positive or uncertain/doubtful). For the purposes of this study, all positive findings, regardless of the assigned certainty, were considered positive. Only bounding boxes generated for the presence of bone fractures were considered.

### Data analysis—diagnostic accuracy

This study collected four sets of results for the identification of fractures: (1) a “ground truth” defined by agreement between two consultant paediatric radiologists and used as a reference to assess the correctness of the other results, (2) the AI diagnoses alone, (3) the first diagnosis round by radiologists without AI assistance, and (4) the second diagnosis round by radiologists with AI assistance. After both rounds were completed, the results from radiologists without AI assistance, radiologists with AI assistance, and the AI alone were compared against the ground truth by calculating the intersection between the bounding boxes. Overlap of radiologist, or AI, and ground truth bounding boxes by at least 40% was considered a true positive; less than 40% was considered a false positive. Failure to place a bounding box on an image where a fracture was present was considered a false negative. Details on how the threshold of 40% was decided for the intersection are provided in the Electronic Supplementary Material, including information on what is meant by overlap.

The results were computed at a per-fracture, per-image and per-examination level for each radiologist.

- At the per-fracture level, correct identification of a fracture was simplified to any bounding box drawn by a radiologist that has over 40% overlap with a gold standard bounding box (most strict definition of fracture detection).
- At the per-image level, any image upon which a radiologist drew a bounding box was counted as positive, and correctness was determined by assessing whether that image contained at least one fracture bounding box from ground truth, irrespective of accurate location or number of fractures.
- At the per-examination level, examinations were determined positive by radiologists when an examination had at least one image classified as positive by that radiologist, irrespective of correct projection or image (loosest and most generous definition of fracture detection).

The confidence intervals for diagnostic accuracy, sensitivity, and specificity were “exact” Clopper-Pearson confidence intervals [16], while the PPV and NPV used a variation of the Wilson confidence interval [17] computed using the efficient-score method developed by Robert Newcombe [18], at each of the three levels of results.

### Inter and intra-reader variability

Intra-reader agreement was assessed through the changes in diagnoses made by a radiologist between rounds 1 and 2.

At the per-examination and per-image level, there is a consistent number of examinations and images for each radiologist between rounds, enabling a direct comparison. We further analysed the changes by assessing whether a change was from a positive diagnosis to a negative diagnosis or vice versa and whether that change was made in alignment with the result from the AI (meaning the radiologist’s classification in round 2 was the same as the AI).

The per-fracture level has an inconsistent number of results for each radiologist between rounds, impeding the ability to conduct this analysis in the same manner. Therefore, changes to false positive fracture identifications were only considered when the amount within an image changed between rounds, with each added or removed false positive considered one change from or to a true negative, respectively.

The correlation between radiologists’ results were calculated at the per examination and per image level using the implementation of Cohen’s Kappa in the Python SciPy Metrics library [19], as per the method suggested by Hallgren [20] suggesting computing the Cohen’s Kappa for each reader-pair and taking the arithmetic mean to arrive at a single value for measuring the inter-reader agreement. The same function was used at a per-fracture level with the adjustment that only the first instance of false-positive fracture within an image was considered, due to the same limitation as when calculating the per-fracture intra-reader agreement.

The agreement between the radiologists and the AI was determined in a similar method, taking the mean Cohen’s Kappa for each radiologist with AI.

### Failure analysis

Cases where a majority, defined as five or more radiologists, initially detected either the presence or absence of a fracture differently to the AI were of particular interest and isolated to provide a clearer understanding of the relative sensitivities.

These were identified through the comparison of predictions from radiologists in round 1 versus the AI acting standalone, at per-fracture level.

Instances where the AI was correct and five or more radiologists were incorrect, and vice versa, were counted, with the limitation that false positives were not required to be in the same location. Changes radiologists made to their diagnosis of an image between rounds were also analysed further to identify any trends in AI influence on radiologists’ decision-making processes.

## Results

### Dataset

In total, across the 13-month retrospective study review period, 48 patients (39/48, 81.2% male, average age: 12 years (range: 6–17 years, s.d.: 3.5 years)) were identified for inclusion. All available examinations of these patients at their appointment(s) during this study period were collated to form a dataset of 336 unique image projections, grouped into 107 appendicular and pelvic radiographic examinations (6/107, 5.6% upper limb radiographs; 93/107, 86.9% lower limb radiographs; 8/107, 7.5% pelvic radiographs).

A total of 206 fractures were identified in the entire dataset as the ground truth. Of the total images, 148/336 (44%) were annotated with at least one acute or healing fracture bounding box. Out of the 107 examinations, 65 (61%) included at least one image that contained a labelled fracture. The ground truth identified 40/206 (19.4%) fractures as acute, with the remaining 166/206 (80.6%) fractures labelled as healing.

**Fig. 1:**
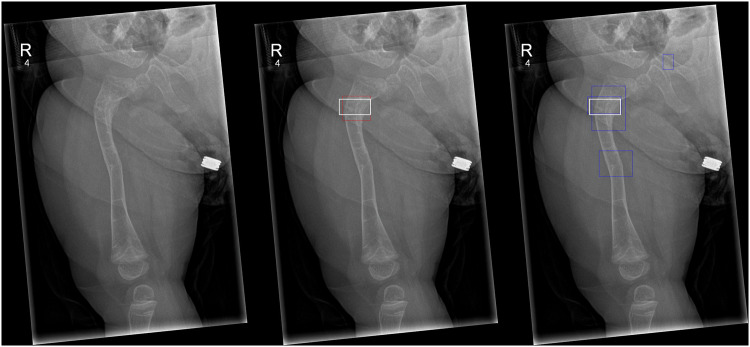
Frontal radiographic projection of the right femur in a female patient aged 1 year, demonstrating one acute fracture at the femoral neck. In this case, the AI correctly identified a fracture, which was missed by 5 or more radiologists. Left: Unmarked radiograph. Middle: Radiograph with AI detection indicated by a red box and ground truth by the white box. Right: Radiograph with radiologists’ detections indicated in blue boxes and ground truth in white box. Two radiologists correctly identified the fracture

**Fig. 2:**
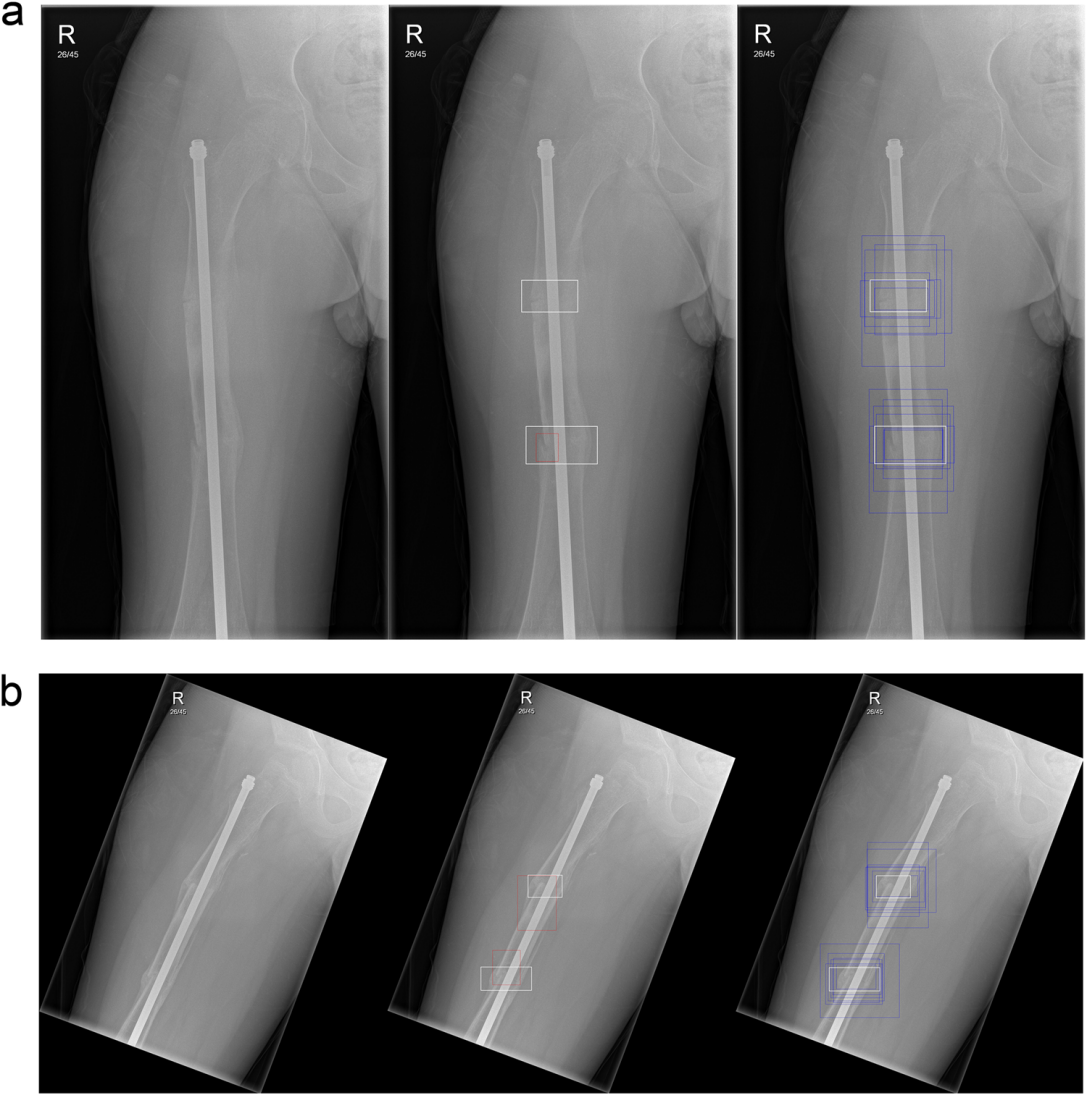
**a** Frontal radiographic projection of the right femur, with an intramedullary rod in situ, in a male patient aged 11 years old, demonstrating two healing fractures, one at the proximal femoral diaphysis, another at the mid-diaphysis region. In this case, almost all radiologists correctly identified both fractures; however, the AI only detected one fracture. Of note, in this case, the radiologists did not change their answers from the first round to the second round for their findings. Left: Unmarked radiograph. Middle: Radiograph with AI detection indicated by red boxes and ground truth label by white box. Right: Radiograph with different radiologists’ fracture annotations indicated by blue boxes and ground truth by the white box. **b** Lateral projection of the right femur in the same patient. Left: Unmarked radiograph. Middle: Radiograph with ground truth labelled by a white box. Right: Radiograph with radiologists’ detections indicated by blue boxes and the ground truth labelled by a white box

**Fig. 3:**
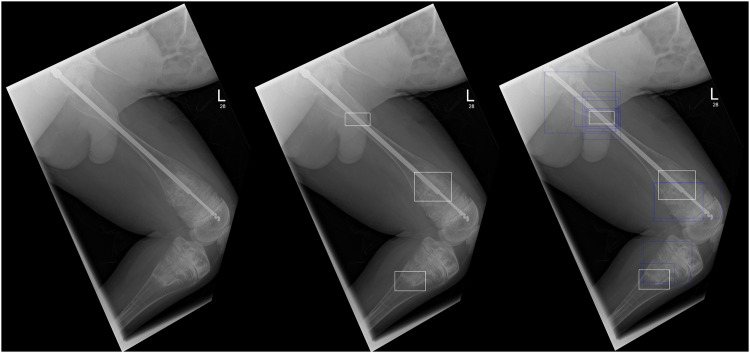
Lateral projection of the left femur in a male patient aged 15 years, with three fractures—healing proximal femoral diaphyseal fracture, acute distal femoral metaphyseal fracture and acute proximal tibial fracture. In this example, the majority of radiologists correctly identified the proximal femoral diaphyseal fracture, with a few radiologists correctly identifying the other two fractures. The AI did not identify any of the fractures. In this particular case, some radiologists did amend their (positive) answers for the distal femoral and proximal tibial fractures to no fracture. Left: Unmarked radiograph. Middle: Radiograph ground truth annotations indicated by white boxes (no AI detection). Right: Radiograph with radiologists’ detections indicated by blue boxes and ground truth by white boxes

**Fig. 4:**
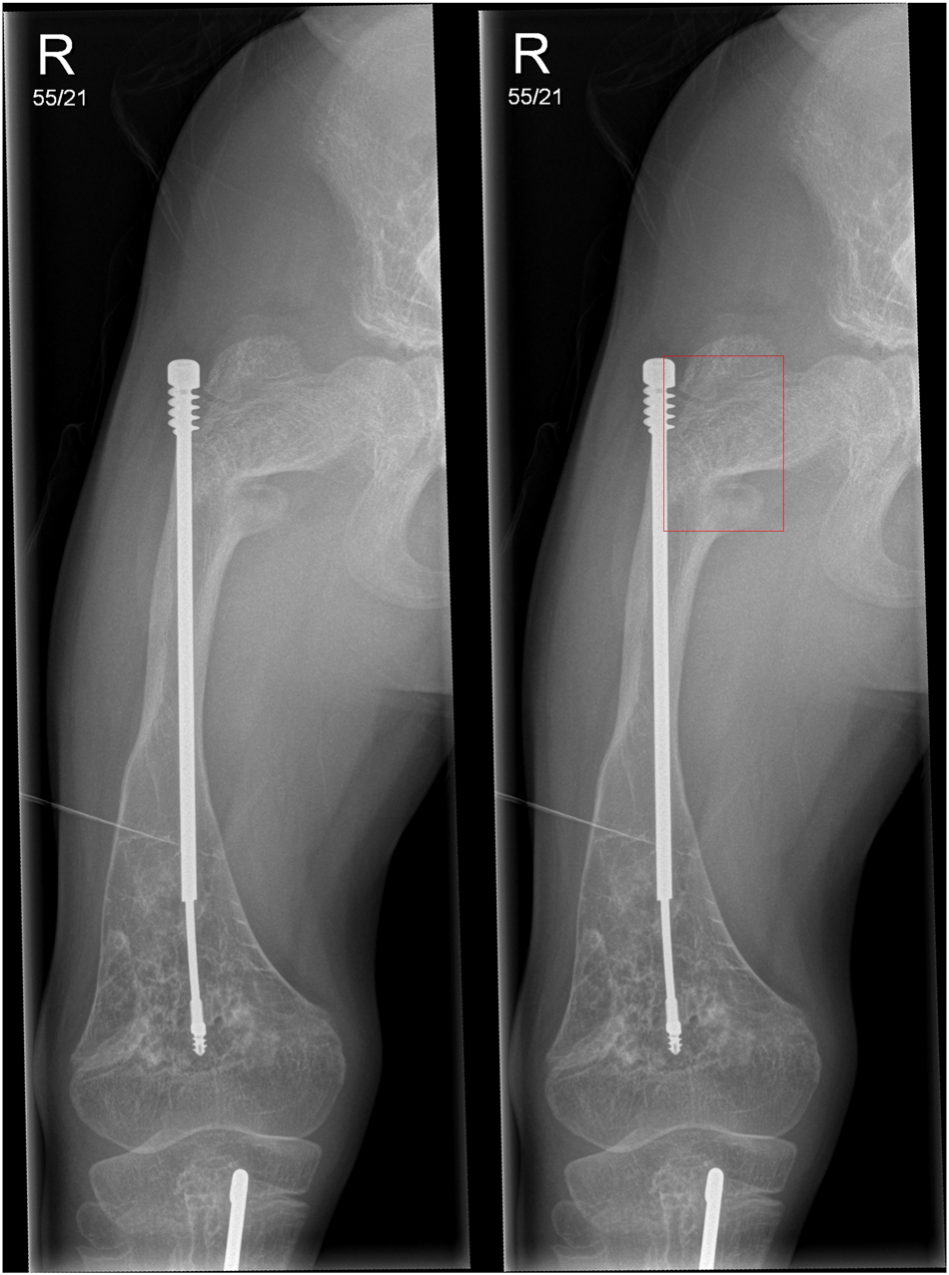
Frontal projection of the right femur with an intramedullary rod, without any acute or healing fractures, in a male patient aged 12 years old. In this example, none of the radiologists detected a fracture on the radiograph; however, the AI produced a false positive detection for a fracture (red box) at the physis of the greater trochanter. Radiologists were confident in their decision and did not change their answers from the first round to the second round. Left: Unmarked radiograph. Right: Radiograph with AI detection indicated by red box

The information on the distribution of the dataset is summarised in Table 1. Example images of differences in fracture identification between radiologists and AI are demonstrated in Figs. 1–4.

**Table 1:** Table indicating the distribution of patients, examinations, images, and fractures in the dataset

	Male	Female	Total
Patients	39	9	48

	Fracture present	No fracture present	Total
Examinations	65	42	107
Images	148	188	336

	Acute	Healing	Total
Fractures	40	166	206

### Overall outcomes

**Fig. 5:**
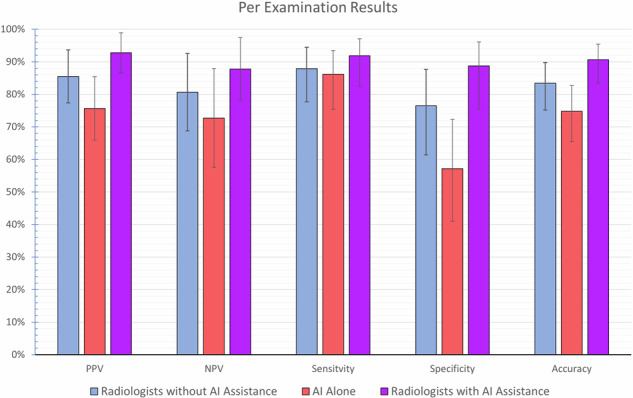
Bar graph visualising the per-examination results for positive predictive value (PPV), negative predictive value (NPV), sensitivity, specificity, and accuracy. The confidence intervals for each metric are represented by error bars

Overall, radiologists performed better than AI alone in fracture detection in this population, with a per-examination accuracy of 83.4% [95% CI: 75.2%, 89.8%] versus 74.8% [95% CI: 65.4%, 82.7%], respectively. However, radiologists using AI assistance resulted in a significant improvement over radiologists acting alone, with average accuracy per examination increasing by 7.3% to 90.7% [95% CI: 83.5%, 95.4%]. This increase was also shown on a per image basis, with accuracy increasing by 7.0% (84.6% [95% CI: 80.3%, 88.2%] versus 91.6% [95% CI: 88.2%, 94.3%]), and a per fracture basis with accuracy increasing by 3.7% (76.3% [95% CI: 72.0%, 80.3%] versus 80.0% [95% CI: 75.9%, 83.7%]) (Table 2). The results, per examination, are further visualised in Fig. 5.

**Table 2:** Breakdown of performance per examination/image/fracture, for radiologists without AI, with AI and AI only

Per examination	Avg. radiologist	Avg. radiologist + AI	Δ	AI alone	Avg. radiologist Δ	Avg. radiologist + AI Δ
True positive	57.1	59.7	2.6	56.0	−1.1	−3.7
False positive	9.9	4.7	−5.1	18.0	8.1	13.3
True negative	32.1	37.3	5.1	24.0	−8.1	−13.3
False negative	7.9	5.3	−2.6	9.0	1.1	3.7
PPV	85.5%	92.8%	7.3%	75.7%	−9.8%	−17.1%
NPV	80.7%	87.7%	7.1%	72.7%	−7.9%	−15.0%
Sensitivity	87.9%	91.9%	4.0%	86.2%	−1.8%	−5.7%
Specificity	76.5%	88.8%	12.2%	57.1%	−19.4%	−31.6%
Accuracy	83.4%	90.7%	7.2%	74.8%	−8.7%	−15.9%

Per image	Avg. radiologist	Avg. radiologist + AI	Δ	AI alone	Avg. radiologist Δ	Avg. radiologist + AI Δ
True positive	127.9	133.4	5.6	119.0	−8.9	−14.4
False positive	31.7	13.6	−18.1	31.0	−0.7	17.4
True negative	156.3	174.4	18.1	157.0	0.7	−17.4
False negative	20.1	14.6	−5.6	29.0	8.9	14.4
PPV	80.8%	91.1%	10.3%	79.3%	−1.5%	−11.8%
NPV	88.8%	92.4%	3.6%	84.4%	−4.4%	−8.0%
Sensitivity	86.4%	90.2%	3.8%	80.4%	−6.0%	−9.8%
Specificity	83.1%	92.8%	9.7%	83.5%	0.4%	−9.3%
Accuracy	84.6%	91.6%	7.1%	82.1%	−2.4%	−9.5%

Per fracture	Avg. radiologist	Avg. radiologist + AI	Δ	AI alone	Avg. radiologist Δ	Avg. radiologist + AI Δ
True positive	164.6	158.9	−5.7	126.0	−38.6	−32.9
False positive	58.6	36.4	−22.1	62.0	3.4	25.6
True negative	156.3	174.4	18.1	140.0	−16.3	−34.4
False negative	41.4	47.1	5.7	80.0	38.6	32.9
PPV	74.6%	81.8%	7.2%	67.0%	−7.5%	−14.7%
NPV	79.3%	79.0%	−0.4%	63.6%	−15.7%	−15.3%
Sensitivity	79.9%	77.1%	−2.8%	61.2%	−18.7%	−16.0%
Specificity	73.1%	83.0%	10.0%	69.3%	−3.8%	−13.7%
Accuracy	76.3%	80.0%	3.7%	65.2%	−11.1%	−14.8%

Columns labelled ‘Δ’ denote changes in numbers/performance

At the per-fracture level, the number of true positives (TP) and false positives (FP) both decreased, whereas the number of true negatives (TN) and false negatives (FN) both increased from round 1 to 2. This resulted in the overall accuracy, specificity, and PPV increasing by 3.7%, 10.0% and 7.2%, respectively, by radiologists together with AI and sensitivity decreasing by 2.8%, and NPV by 0.4%.

### Acute versus healing fracture types

Although the AI did not provide information related to fracture healing, overall radiologists demonstrated a higher accuracy for classifying fracture type with AI assistance (73.3% [95% CI: 68.9%, 77.4%]) than alone (68.2% [95% CI: 63.6%, 72.5%]) (Table S1 in Supplementary Material).

### Changes in decision making

Changes to fracture detection between the two rounds on a per-fracture, per-image and per-examination level are shown in Table 3. Radiologists, by arithmetic mean, at a per-fracture level altered their original decision in 72 events, of which 69% (49/72) were aligned with the AI output. Predominantly, these changes were the correct ones to make, with approximately 64% leading to a more accurate result (e.g., either TN or TP). Most of the changes by the radiologists were from a positive finding of a fracture to a negative one, accounting for 71% of changes at the per-fracture level.

**Table 3:** Average number of changes in classification of fractures from Round 1 to Round 2 made by radiologists and whether these resulted in correct or incorrect decisions, with subgrouping as to whether these were in accordance with or not with the AI assistance in Round 2

	Per examination (of 107 cases overall)		Per image (of 336 images overall)		Per fracture (of 394 fracture events)	
	*n*/total	%	*n*/total	%	*n*/total	%
Overall average number of changes	18.00/107.00	16.82%	50.00/336.00	14.88%	71.86/394.00	18.24%
Correct change	12.86/18.00	71.43%	36.86/50.00	73.71%	40.71/71.86	56.66%
Wrong change	5.14/18.00	28.56%	13.14/50.00	26.28%	31.14/71.86	43.33%
Subgroup: Positive → Negative	10.29/18.00	57.14%	31.29/50.00	62.57%	47.86/71.86	66.60%
Correct change	7.00/10.29	68.06%	22.86/31.29	73.06%	22.86/47.86	47.76%
Wrong change	3.29/10.29	31.97%	8.43/31.29	26.94%	25.00/47.86	52.24%
Subgroup: Negative → Positive	7.71/18.00	42.86%	18.71/50.00	37.43%	24.00/71.86	33.40%
Correct change	5.86/7.71	75.93%	14.00/18.71	74.81%	19.29/24.00	80.36%
Wrong change	1.86/7.71	24.12%	4.71/18.71	25.17%	4.714/24.00	19.64%
Disagreement with AI	5.71/18.00	31.72%	12.71/50.00	25.42%	22.43/71.86	31.19%
Correct change	4.00/5.71	70.05%	6.00/12.71	47.19%	9.14/22.43	40.76%
Wrong change	1.71/5.71	29.94%	6.71/12.71	52.79%	13.29/22.43	59.25%
Subgroup: Positive → Negative	4.71/5.71	82.50%	7.43/12.71	58.43%	12.57/22.43	56.05%
Correct change	3.29/4.71	69.70%	3.86/7.43	51.92%	3.86/12.57	30.68%
Wrong change	1.43/4.71	30.36%	3.57/7.43	48.05%	8.71/12.57	69.29%
Subgroup: Negative → Positive	1.00/5.71	17.50%	5.29/12.71	41.57%	9.86/22.43	43.95%
Correct change	0.71/1.00	71.43%	2.14/5.29	40.54%	6.71/9.86	68.12%
Wrong change	0.29/1.00	29.00%	3.14/5.29	59.36%	3.14/9.86	31.85%
Agreement with AI	12.29/18.00	68.25%	37.29/50.00	74.57%	49.43/71.86	68.79%
Overall correct change	8.86/12.29	72.09%	30.86/37.29	82.76%	31.57/49.43	63.87%
Overall wrong change	3.43/12.29	27.90%	6.43/37.29	17.24%	17.86/49.43	36.13%
Subgroup: Positive → Negative	5.57/12.29	45.35%	23.86/37.29	63.98%	35.29/49.43	71.39%
Correct change	3.71/5.57	66.67%	19.00/23.86	79.64%	19.00/35.29	53.85%
Wrong change	1.85/5.57	33.21%	4.86/23.86	20.37%	16.29/35.29	46.16%
Subgroup: Negative → Positive	6.71/12.29	54.65%	13.43/37.29	36.02%	14.14/49.43	28.61%
Correct change	5.14/6.71	76.60%	11.86/13.43	88.30%	12.57/14.14	88.89%
Wrong change	1.57/6.71	23.39%	1.57/13.43	11.69%	1.57/14.14	11.10%

“Correct change” means a change in decision which resulted in an accurate outcome (aligned with ground truth). Incorrect change means a change in decision which led to an inaccurate outcome (at odds with the ground truth). Positive to negative means identification of a fracture (+), which was then changed to no fracture (−). There are more “fracture events” than actual fractures in the dataset to account for all the times a radiologist classified a fracture (of which some were overcalls)

### Inter-reader agreement

With AI assistance, there was a significant increase in the inter-reader agreement on a per-examination (0.52 to 0.74), per-image (0.57 to 0.78) and per-fracture level (0.53 to 0.66) (Table S2 in Supplementary Material). The level of agreement between radiologists using AI at round 2 was higher than between radiologists in round 1 without AI assistance.

### Failure analysis

Table 4 demonstrates the total occurrences where 5 or more radiologists disagreed with the AI and in which situations they were correct or incorrect (according to ground truth). Overall, there were 86 instances of majority disagreement between radiologists and AI, and of these 76/86 (88%) the radiologists were correct and the AI incorrect.

**Table 4:** Summary of individual situations where the majority of radiologists contradicted the AI, and for which they were correct versus the AI (compared to our ground truth)

	Number of radiologists			Total	
	5	6	7		
AI correct					
Radiologist overcall (FP)	2	5	0	7	10
Radiologist undercall (FN)	3	0	0	3	
Radiologist correct					
AI overcall (FP)	9	5	10	24	76
AI undercall (FN)	16	20	16	52	

The AI was correct in 10 situations where the majority of radiologists were incorrect (most of these being overalls by the radiologists). The majority of radiologists were correct in 76 situations where the AI was incorrect (most of these being misses by the AI)

In the majority of cases where AI was incorrect, this was due to a miss/undercall (i.e., false negative finding) in 68% of cases. Conversely, when the AI produced a correct diagnosis and five or more radiologists were incorrect (*n* = 10), radiologists were over-calling (i.e., false positive) in the majority (7/10, 70%).

## Discussion

In this study, we found that radiologists performed better than AI in detecting fractures in children with OI, but radiologists assisted by AI performed better than those reporting alone. When radiologists amended their original decision to align with those of the AI, it was the correct decision in the majority of cases. There was a greater inter-reader agreement after AI assistance, confirming that the AI acts as a common reference for all radiologists and leads to more consistent diagnoses across the board.

In this cohort of children with OI, the diagnostic accuracy score for the AI tool was much lower than expected, even when calculating this on a per-examination level (accuracy rate of 75%, sensitivity 86%, specificity 57%). The same commercially available AI product has a reported and published standalone diagnostic accuracy for fracture detection in adults of 90.1% (sensitivity of 90.2%, specificity of 92.5%) [13], and receiver operating characteristic area under curve of 95.8% (sensitivity of 91.9%, specificity of 87.8%) in another study [14]. In a cohort of 478 children (aged 6–13 years old, mean age 10 years), the AI tool achieved a comparable standalone receiver operating characteristic area under curve to adults of 95.4% (sensitivity of 93.6%, specificity of 84.6%) [14]. We are, however, unaware of any studies evaluating the use of any AI tool for fracture detection in a population of patients (regardless of age) with OI, despite their increased bone fragility, greater likelihood for delayed diagnosis [21] and reduced quality of life following multiple injuries [22, 23]. We conclude that the reduction in fracture detection and accuracy is likely due to this population, which underlines the need for studies evaluating patients with rare diseases, but at high risk of pathology, and emphasises how indiscriminate deployment of algorithms trained in one population may differ when applied to another different population. Given the limited number of cases for analysis and review, we were unable to yield any meaningful subgroup analysis to understand if this reduction in AI performance was particularly worse for certain subtypes of OI or age groups; however, this would be interesting to evaluate in a larger cohort.

Interestingly, despite the poorer standalone performance of the AI compared to that of the radiologists, we found an overall beneficial effect when AI assistance was provided. In our study, radiologists typically overcalled findings on the radiographs (FP), which may be related to the perceived greater consequences of missing a fracture in this patient population. Conversely, the AI tool was more likely to undercall (i.e., miss) abnormalities, which may reflect neutrality regarding the population and unfamiliarity with some of the abnormal appearances, which contributed to its poorer standalone performance. Together, this under-calling had a surprising moderating effect by reducing the radiologists’ false positive rates. Despite the moderation, radiologists still frequently overruled the AI in most false-negative cases. This is reflected in the observed decrease in sensitivity and NPV, accompanied by an increase in specificity and PPV. This finding contrasts with other AI-assisted radiologist studies where performance increased but where the AI systems were always at least as good or better than that of human radiologists [9]. We should however be mindful of the fact that all our radiologists in this study were either experienced paediatric radiologists or senior trainees with at least 6 months of paediatric radiology experience and therefore may be more confident in overruling the AI in false negative cases than non-specialist reporters or non-radiologists.

Concerns about deskilling and automation bias have become prominent in the AI community, revolving around the potential decrease in human vigilance and confidence in overriding AI systems [24]. While these fears are legitimate, our study (acknowledging this was conducted in a controlled environment where radiologists were aware of being observed) demonstrated that a human in the loop could effectively collaborate with AI tools (understanding when the AI performs poorly) and result in improved overall accuracy. Our results therefore underscore the critical need for human oversight when integrating new AI tools and not to ignore proper radiographic training, especially when the diagnostic limitations may not be fully understood with some rare diseases at early AI implementation.

Several limitations to this study are noted. Given the rarity of the disease being studied and the desire to maximise available imaging for review, we included repeat examinations from some patients over the study period and considered them as separate subjects for simplicity of analysis. The radiologists were not made aware of the repeated studies, but inherent similarities in appearances may have biased their assessment of the radiographs and led to an imbalance in the distribution of fracture types and anatomical regions for review. For this reason, we also did not share prior images for comparison with the radiologist, even though this would reflect normal clinical practice. We only used one commercially available AI model, even though this tool currently outperforms other commercial products on the market for fracture detection [13]. Further work evaluating unique examinations from a larger dataset across multiple international centres, and across different available AI models, would help improve the generalisability of our results.

To objectively evaluate whether AI tools perform less accurately in children with OI, a control group of patients without a bone fragility disorder would have been an ideal comparator. Nevertheless, matching fracture sites, body parts, age and gender, poses a considerable challenge for such a study given that patients with OI fracture in unusual locations that are not easily matched in a “healthy” cohort, e.g., mid-femoral fractures are not typically seen outside of major trauma in healthy children [25].

Finally, we tried to minimise radiologist’s recall bias by using a washout period and randomising the order of the images at round 2; however, this does not necessarily remove the possibility that radiologists’ diagnostic performance could have improved over time. Future studies that include one or more control radiologists, who do not use AI assistance in either round, or use AI assistance at the first round instead of the second, may provide a useful comparator.

In conclusion, the results of this study suggest that AI assistance improves radiologists’ performance in diagnosing fractures in children with OI, even if it is not specifically trained for this population. In our study, the AI tool moderated the number of overcalls by radiologists and led to more consistency in diagnostic outcomes (through higher inter-reader agreement), without necessarily impeding radiologist’s ability to overrule the AI when it misses obvious fractures. Nevertheless, compared to radiologists, the standalone AI performance was worse, thus highlighting potential dangers of implementing the AI tool in an autonomous manner. Future studies focusing on fine-tuning and evaluating fracture detection tools specifically for children and those with rare diseases are vital to ensure maximal societal benefit and health equity from digital innovation.

## Supplementary information

ELECTRONIC SUPPLEMENTARY MATERIAL

## Abbreviations

AI: Artificial intelligence
NHS: National Health Service
NPV: Negative predictive value
OI: Osteogenesis imperfecta
PPV: Positive predictive value

## References

[1] Warman ML, Cormier-Daire V, Hall C et al (2011) Nosology and classification of genetic skeletal disorders: 2010 revision. Am J Med Genet A 155A:943–968 21438135 10.1002/ajmg.a.33909 PMC3166781

[2] Sillence DO, Senn A, Danks DM (1979) Genetic heterogeneity in osteogenesis imperfecta. J Med Genet 16:101–116 458828 10.1136/jmg.16.2.101 PMC1012733

[3] Zhao X, Yan SG (2011) Recent progress in osteogenesis imperfecta. Orthop Surg 3:127–130 22009598 10.1111/j.1757-7861.2011.00128.x PMC6583639

[4] Sharma S (2023) Artificial intelligence for fracture diagnosis in orthopedic X-rays: current developments and future potential. SICOT J 9:21 37409882 10.1051/sicotj/2023018 PMC10324466

[5] Zech JR, Santomartino SM, Yi PH (2022) Artificial intelligence (AI) for fracture diagnosis: an overview of current products and considerations for clinical adoption, from the AJR Special Series on AI Applications. AJR Am J Roentgenol 219:869–878 35731103 10.2214/AJR.22.27873

[6] Shelmerdine SC, White RD, Liu H, Arthurs OJ, Sebire NJ (2022) Artificial intelligence for radiological paediatric fracture assessment: a systematic review. Insights Imaging 13:94 35657439 10.1186/s13244-022-01234-3 PMC9166920

[7] Rainey C, McConnell J, Hughes C, Bond R, McFadden S (2021) Artificial intelligence for diagnosis of fractures on plain radiographs: a scoping review of current literature. Intell-Based Med 5:100033

[8] Pishtiwan HSK, Sebastian S, Sergey P et al (2020) Deep learning in fracture detection: a narrative review. Acta Orthop 91:215–220 31928116 10.1080/17453674.2019.1711323 PMC7144272

[9] Kuo RYL, Harrison C, Curran TA et al (2022) Artificial intelligence in fracture detection: a systematic review and meta-analysis. Radiology 304:50–62 35348381 10.1148/radiol.211785 PMC9270679

[10] Lee J, Liu C, Kim J et al (2022) Deep learning for rare disease: a scoping review. J Biomed Inform 135:104–227 10.1016/j.jbi.2022.104227 36257483

[11] Visibelli A, Roncaglia B, Spiga O, Santucci A (2023) The impact of artificial intelligence in the odyssey of rare diseases. Biomedicines 11:887 10.3390/biomedicines11030887 PMC10045927 36979866

[12] Tejani AS, Klontzas ME, Gatti AA et al (2024) Checklist for artificial intelligence in medical imaging (CLAIM): 2024 update. Radiol Artif Intell 6:e240300 38809149 10.1148/ryai.240300 PMC11304031

[13] Bousson V, Attané G, Benoist N et al (2023) Artificial intelligence for detecting acute fractures in patients admitted to an emergency department: real-life performance of three commercial algorithms. Acad Radiol 30:2118–2139 37468377 10.1016/j.acra.2023.06.016

[14] Parpaleix A, Parsy C, Cordari M, Mejdoubi M (2023) Assessment of a combined musculoskeletal and chest deep learning-based detection solution in an emergency setting. Eur J Radiol Open 10:100482 36941993 10.1016/j.ejro.2023.100482 PMC10023863

[15] Shelmerdine SC, Martin H, Shirodkar K, Shamshuddin S, Weir-McCall JR (2022) Can artificial intelligence pass the Fellowship of the Royal College of Radiologists examination? Multi-reader diagnostic accuracy study. BMJ 379:e072826 36543352 10.1136/bmj-2022-072826 PMC9768816

[16] Clopper CJ, Pearson ES (1934) The use of confidence or fiducial limits illustrated in the case of the binomial. Biometrika 26:404–413

[17] Wilson EB (1927) Probable inference, the law of succession, and statistical inference. J Am Stat Assoc 22:209–212

[18] Newcombe RG (1998) Two-sided confidence intervals for the single proportion: comparison of seven methods. Stat Med 17:857–872 9595616 10.1002/(sici)1097-0258(19980430)17:8<857::aid-sim777>3.0.co;2-e

[19] Virtanen P, Gommers R, Oliphant TE et al (2020) SciPy 1.0: fundamental algorithms for scientific computing in Python. Nat Methods 17:261–272 32015543 10.1038/s41592-019-0686-2 PMC7056644

[20] Hallgren KA (2012) Computing inter-rater reliability for observational data: an overview and tutorial. Tutor Quant Methods Psychol 8:23–34 22833776 10.20982/tqmp.08.1.p023 PMC3402032

[21] Malina SN, Flanagan JC, Loechner KJ, Wu M (2023) Access to care among patients with osteogenesis imperfecta during the COVID-19 pandemic. Arch Osteoporos 18:143 38015270 10.1007/s11657-023-01355-2 PMC10924804

[22] Flanagan J, Tosi L, Carter E, Hart T, Franzone J, Wallace M (2023) “Osteogenesis imperfecta patients wish orthopedic surgeons had better strategies to help with…”—results of a patient and parent-oriented survey. Children (Basel) 10:1345 10.3390/children10081345 PMC10453135 37628344

[23] Westerheim I, Hart T, van Welzenis T et al (2024) The IMPACT survey: a mixed methods study to understand the experience of children, adolescents and adults with osteogenesis imperfecta and their caregivers. Orphanet J Rare Dis 19:128 38515144 10.1186/s13023-024-03126-9 PMC10956293

[24] Adler-Milstein J, Redelmeier DA, Wachter RM (2024) The limits of clinician vigilance as an AI safety bulwark. JAMA 331:1173–1174 38483397 10.1001/jama.2024.3620

[25] Calder AD (2015) Radiology of osteogenesis imperfecta, rickets and other bony fragility states. Endocr Dev 28:56–71 26138835 10.1159/000380992

