# Supplementary material for: External validation of an artificial intelligence tool for fracture detection in children with osteogenesis imperfecta: a multireader study
Source: Eur Radiol. 2025 Jul 7;36(1):515–25. doi: 10.1007/s00330-025-11790-z (PMC12712034; doi:10.1007/s00330-025-11790-z)
Supplement: Supplementary file 1 — ELECTRONIC SUPPLEMENTARY MATERIAL [file 330_2025_11790_MOESM1_ESM.pdf]

External Validation of an Artificial Intelligence Tool for Fracture Detection in Children with Osteogenesis Imperfecta: A Multireader Study

ELECTRONIC SUPPLEMENTARY MATERIAL

Table of Contents

**Title**	**Page**
Image Acquisition	2
Image Anonymisation and Preprocessing	3
Overlapping ROI boxes	4
Statistical Methods for IoU threshold	5
Figure S1	6
Figure S2a and S2b	7
Table S1	8
Table S2	8
References	8

Supplementary Methodology

Image Acquisition

Imaging of the appendicular skeleton in this study was performed according to local standard imaging protocols on a Siemens Ysio Max radiography machine, using a combination of wall detector and table detector acquisitions.

Depending on the body part imaged, the source to image distance (SID) ranged from 100-180cm. The exposures factors varied depending on the age of the child and body part imaged, as outlined below. Exposure factors for the pelvis were 60-64kV and 2 – 3.2 mAs.

**AGE**	**SHOULDER/ HUMERUS**		**ELBOW**		**WRIST**		**HAND**	
	**kV**	**mAs**	**kV**	**mAs**	**kV**	**mAs**	**kV**	**mAs**
<1 year	60-62	1.6-2	58-62	1.6-2	58-62	1.6-2	58-62	1.6-2
1-5 years	60-63	2-3.2	60-62	2-2.5	60-62	2-2.5	60-62	2-2.5
5-10 years	63	2.5-3.2	62	2-2.5	62	2-2.5	62	2-2.5
10-15 years	63-65	3.2-5	63	2-2.5	63	2-2.5	62	2.5-3.2

**AGE**	**FEMUR**				**KNEE**				**ANKLE**			
	**AP**		**LATERAL**		**AP**		**LATERAL**		**AP**		**LATERAL**	
	**kV**	**mAs**	**kV**	**mAs**	**kV**	**mAs**	**kV**	**mAs**	**kV**	**mAs**	**kV**	**mAs**
<1 year	60-63	3.2	60-63	2-3.2	60-62	2	60-62	2	60-62	2	60-62	2
1-5 years	60-63	2.5-3.2	60-63	2.5-3.2	60-63	2-3.2	60-63	2.5-3.2	60-62	2-2.5	60-62	2-2.5
5-10 years	65	3.2-5	65	3.2-5	62-64	2-3.2	62-64	2-3.2	62-64	2-3.2	62-64	2-3.2
10-15 years	65-70<br>65-70	5-8<br>16-25(G)	65-70	5-8	63-65	2-4	63-65	2-4	63-65	2-4	63-65	2-4

Image Anonymisation and Pre-processing

Radiographic examinations in this study were initially extracted as DICOM files from the hospital PACS system direct onto a password secure hospital research computer containing the FDA and CE regulated imaging viewing software Osirix v12.0 (Pixmeo) (https://www.osirix-viewer.com/) .

From this software DICOM tags that contained any patient identifiable information (e.g. name, hospital number, age, sex, weight, address etc) were removed, leaving only relevant technical information regarding the image and mode of acquisition (e.g. resolution, pixel size) in order to allow for AI analysis.

The anonymised examinations in DICOM format were renamed according to predefined research study numbers before being uploaded to our GDPR compliant imaging multi-reader platform, Collective Minds (details of the platform available here: https://www.applytosupply.digitalmarketplace.service.gov.uk/g-cloud/services/290506033269528)

Overlapping ROI boxes

In drawing the bounding boxes on the image to denote the presence of a fracture, it is important that this is the same area as the region annotated by the ground truth radiologists. In order to do this we needed to compute the degree of 'overlap' between the two boxes and determine how much overlap was allowed in order to be able to say that the correct fracture had been noted by the readers. Ideally this would be 100% overlap, however different readers may draw boxes of different sizes. The methodology for deciding on the 40% limit described in the article methodology is elaborated upon below. An example of what overlap means is demonstrated in the visual below.

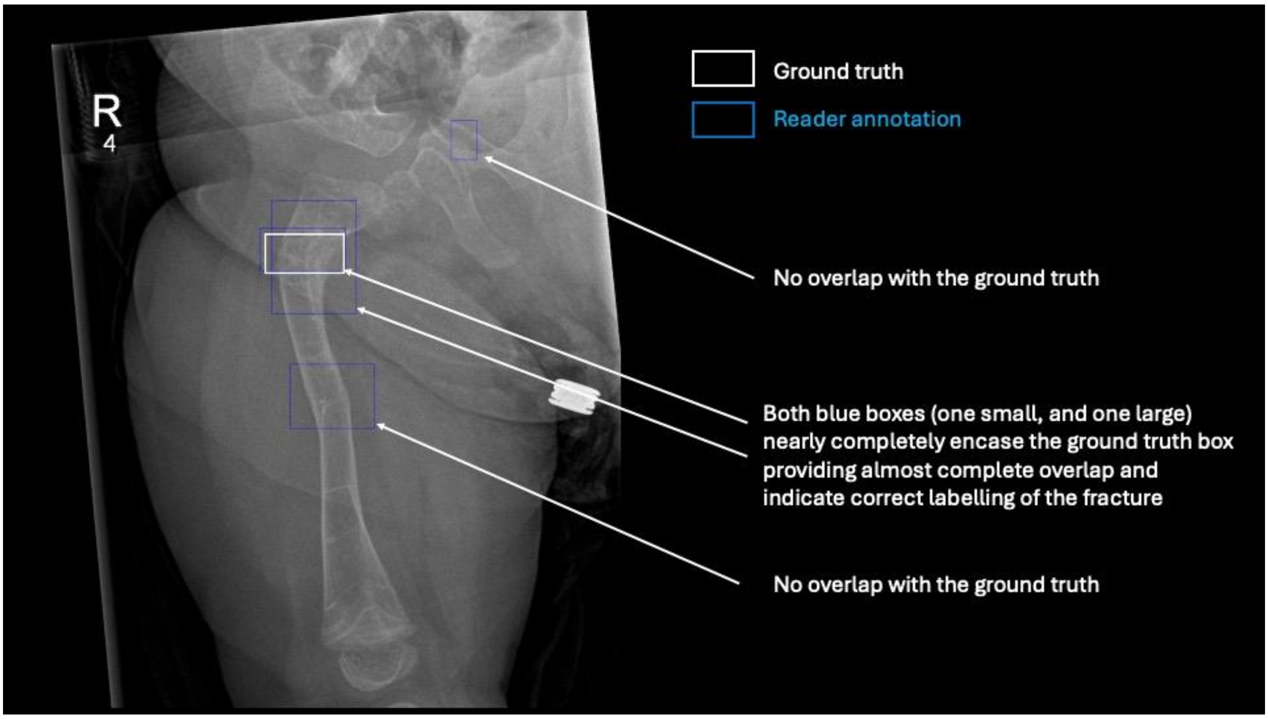

Statistical Methods for IoU threshold

Inconsistencies in the size at which radiologists drew bounding boxes led to the potential for their performance to be incorrectly assessed. We used an adaptation of the Intersection over Union (IoU) metric, which we refer to as the “gold standard intersect”, to compute the ratio of overlap solely in relation to the area of the gold standard bounding box. This metric has a minimum value of 0 when there is no overlap of the radiologists’ and gold standard bounding box and a maximum value of 1 when the radiologists’ bounding box contains the entirety of the gold standard, with no punishment for excessive size. This was considered an acceptable measure to use as there is no risk of the radiologists illegitimately maximising the value.

Multiple bounding boxes on an image, both from radiologists and from the gold standard, were evaluated by computing each possible radiologist and gold standard bounding box pair and selecting the closest matching. We calculated the gold standard intersect for each pair and used Hungarian matching, also known as the Munkres assignment algorithm, to find the optimal bounding box pairs (1). The specific implementation used is the *linear_sum_assignment* function from the Python SciPy Optimize library (2).

After matching the bounding boxes, a threshold was applied to remove radiologists’ bounding boxes that did not reach the minimum gold standard intersect value. These bounding boxes were counted as false positives, as were other predicted bounding boxes that did not have any overlap with a gold standard bounding box. An appropriate threshold of 40% was determined by computing radiologist accuracy at various thresholds, shown in **Figure S1**, and confirmed anecdotally by inspecting random cases close to this threshold, as evidenced in **Figure S2a and S2b**.

We repeated the method for computing bounding box overlap with an adaptation that additionally accounted for correct labelling of fractures. The dataset used included both acute and healing fractures and the gold standard contained labels for categorising each fracture bounding box. In this variation, the labels of each radiologist and gold standard bounding box were first compared. In the case of a mismatch, the gold standard intersect was immediately set to 0, otherwise it was computed as normal. This had the effect of counting any incorrectly labelled bounding boxes as false positives when computing the overall results.

Figure S1. Graph of radiologists' accuracy per fracture, at varying thresholds.

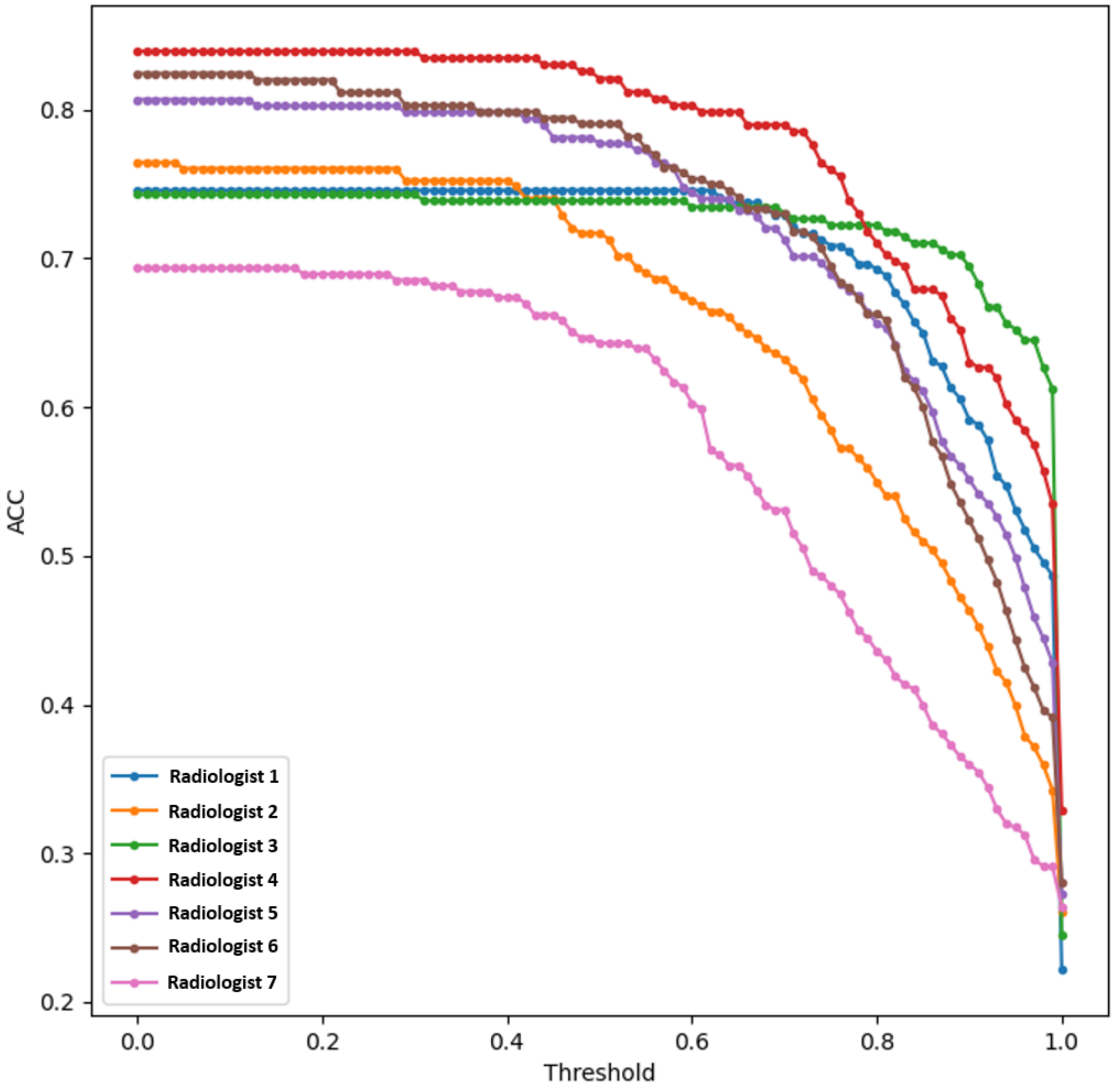

Figure S2a. Two side by side radiographs of the same femur in the same patient. The example on the left is showing the bounding box by the ground truthing radiologists, and the image on the right is demonstrating one of the radiologist's bounding box, with an intersect value of 0.40126 (just above the 40% threshold.

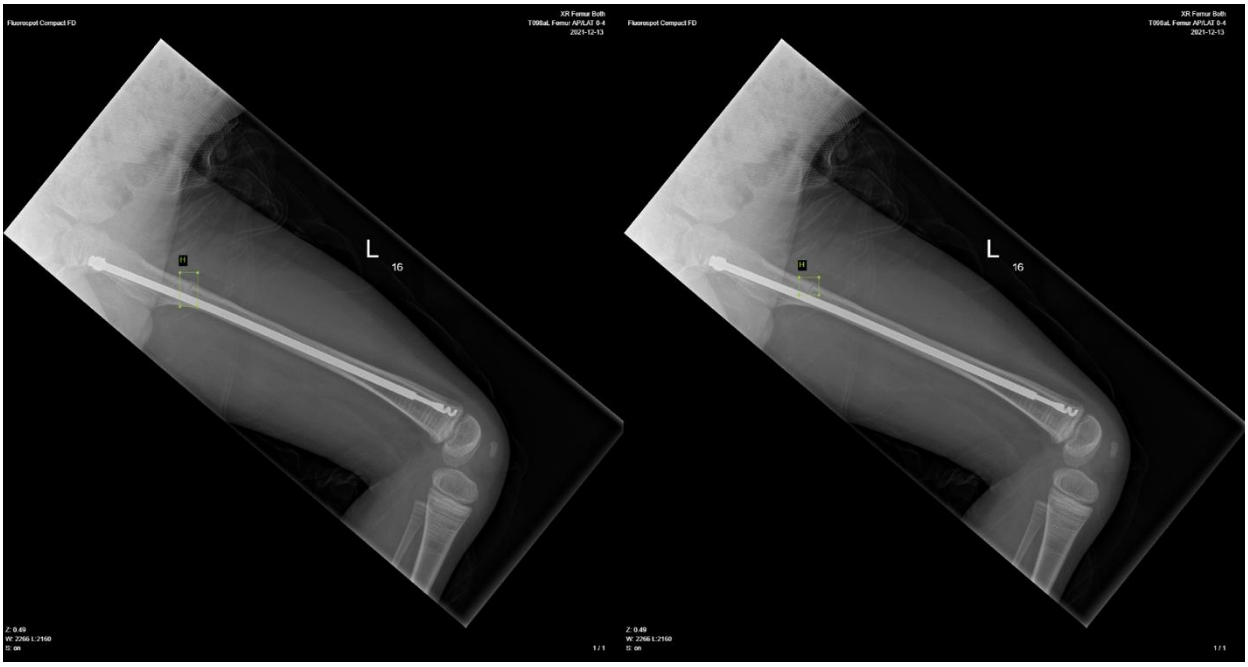

Figure S2b. A magnified version of the two images at the site of fracture are provided below for better visualisation.

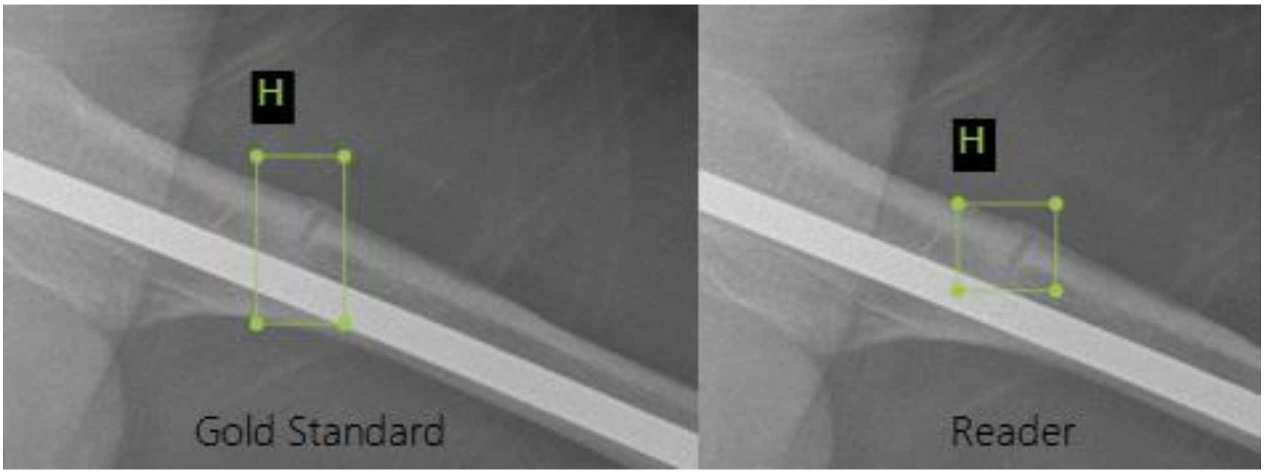

Table S1

Results Per Fracture, for Radiologists Without AI and With AI When Accounting for Correct Acute or Healing Fracture Labelling. Column Labelled 'Δ' Denotes the Changes in Numbers / Performance.

Per Fracture	Radiologists	Radiologists + AI	Δ
TP	144.43	142.71	-1.71
FP	78.71	52.57	-26.14
TN	156.29	174.43	18.14
FN	61.57	63.29	1.71
PPV	65.22%	73.23%	8.01%
NPV	71.89%	73.67%	1.78%
Sensitivity	70.11%	69.28%	-0.83%
Specificity	66.62%	76.99%	10.37%
Accuracy	68.19%	73.29%	5.10%

Table S2

Mean Cohen's Kappa for Each Radiologist Pair for Inter-Reader Correlation and Correlation with AI

	Round 1	Round 2	AI
Per Examination	0.52	0.74	0.54
Per Image	0.57	0.78	0.65
Per Fracture	0.53	0.66	0.52

References:

1. Kuhn HW. The Hungarian Method for the Assignment Problem. In: Jünger M, Liebling TM, Naddef D, Nemhauser GL, Pulleyblank WR, Reinelt G, et al., editors. 50 Years of Integer Programming 1958-2008: From the Early Years to the State-of-the-Art. Berlin, Heidelberg: Springer Berlin Heidelberg; 2010. p. 29-47.

2. Virtanen P, Gommers R, Oliphant TE, Haberland M, Reddy T, Cournapeau D, et al. SciPy 1.0: Fundamental Algorithms for Scientific Computing in Python. Nature Methods. 2020;17(3):261-72.
